# Adenosine stress CMR with variable density spiral pulse sequences accurately detects CAD with minimal dark-rim artifacts

**DOI:** 10.1186/1532-429X-16-S1-O58

**Published:** 2014-01-16

**Authors:** Michael Salerno, Angela M Taylor, Yang Yang, Sujith Kuruvilla, Craig H Meyer, Christopher M Kramer

**Affiliations:** 1 Medicine, Radiology, and BME, University of Virginia, Charlottesville, Virginia, USA; 2 Medicine, Cardiology, University of Virginia, Charlottesville, Virginia, USA; 3 Radiology, University of Virginia, Charlottesville, Virginia, USA; 4 Biomedical Engineering, University of Virginia, Charlottesville, Virginia, USA

## Background

Adenosine stress CMR perfusion imaging has numerous advantages over competing modalities for assessing CAD demonstrating high diagnostic and prognostic utility. However, adenosine stress CMR perfusion imaging is limited by motion-induced dark-rim artifacts (DRA) which may be mistaken for true perfusion abnormalities. We have previously demonstrated that a high-resolution variable-density spiral pulse sequence with a novel density compensation strategy reduces ringing artifacts in first-pass perfusion imaging. The purpose of this study was to assess the clinical performance of this new technique to detect obstructive coronary artery disease (CAD).

## Methods

CMR perfusion imaging was performed during adenosine stress (140 μg/kg-min) and at rest on a Siemens 1.5T Avanto scanner in 41 subjects with chest pain scheduled for coronary angiography (CA). Perfusion images were acquired during injection of 0.1 mmol/kg Gd-DTPA at 3 short-axis locations using a saturation recovery (SR) interleaved variable-density spiral pulse sequence. Sequence parameters included: SR time 80 ms, FOV 320-340 mm2, nominal resolution 2.0 mm2, 8 spiral interleaves, FA 30, TR/TE 9 ms/1 ms. Cine and late gadolinium enhanced (LGE) images were also obtained. All subjects underwent CA following the CMR and significant stenosis was defined as > 50% by quantitative CA. Two blinded reviewers evaluated the spiral perfusion images for the presence of adenosine-induced perfusion abnormalities and assessed image quality using a 5 point scale (1 - poor to 5- excellent).

## Results

**Figure 1:**
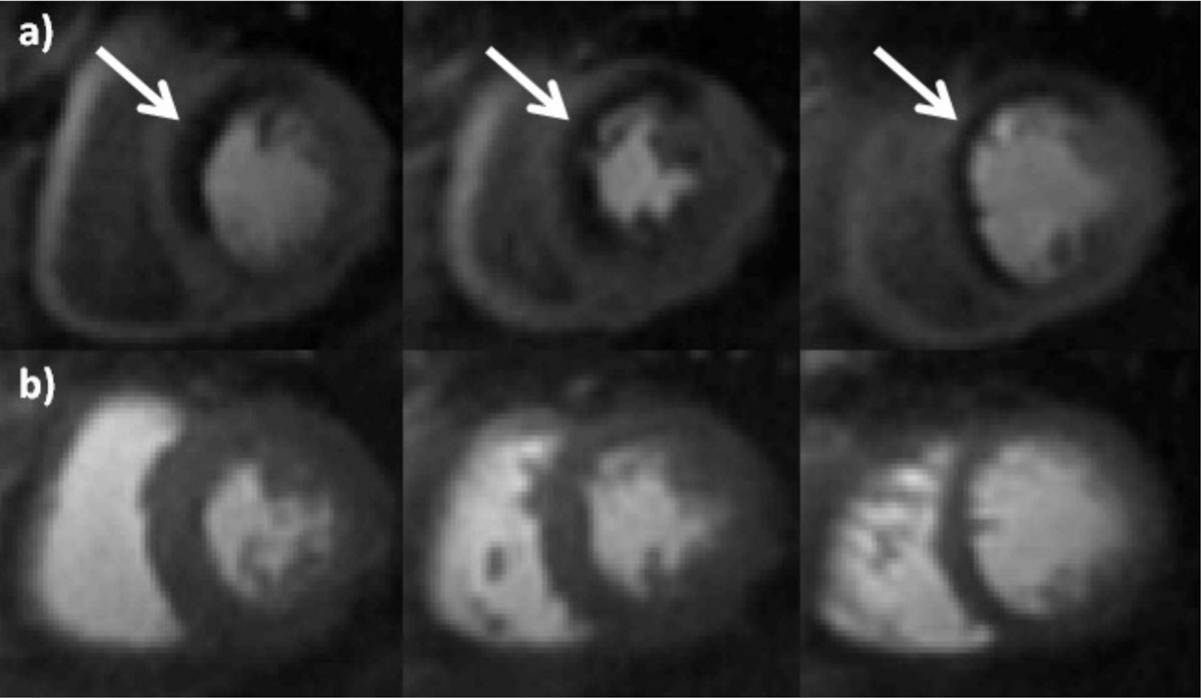
**Stress (a) and Rest (b) adenosine-stress spiral perfusion images demonstrate a large anteroseptal perfusion defect corresponding with a high grade stenosis in the LAD at angiography**. The images have high SNR and resolution with minimal DRA.

The patients had a mean age of 62 ± 9, 68% were male, 51% had a smoking history 46% had diabetes, 78% had hypertension and 95% had hyperlipidemia. 39% of the patients had a known history of CAD and 22% of the patients had undergone prior PCI. The mean LVEF by CMR was 61 ± 7%, and 14 patients (34%) had LGE in a CAD pattern. QCA demonstrated obstructive CAD 28 (68%) of the subjects. Figure 1 shows stress and rest spiral perfusion images from a subject who had normal cardiac function and no LGE. A reversible perfusion defect is present in the anterior wall corresponding to a high grade lesion in the LAD at CA. For the detection of a 50% stenosis by QCA the average sensitivity, specificity, and accuracy of the two readers were 89.3%, 84.6%, and 87.8% respectively with a positive predictive value and negative predictive value of 92.6% and 79% respectively. The average image quality score was 4.4 ± 0.7 with only one study with more than mild DRA. There was good inter-reader reliability with a kappa statistic of 0.67.

## Conclusions

This study is the first to clinically evaluate spiral pulse sequences for adenosine stress CMR. There are a number of advantages to spiral pulse sequences including high efficiency, high SNR efficiency, robustness to motion, and isotropic spatial resolution. We demonstrate that these sequences produce high quality images with minimal dark-rim artifacts and demonstrate high diagnostic accuracy for assessment of CAD.

## Funding

AHA 10SDG2650038, NIH K23 HL112910-01, Siemens Medical Solutions.

